# Synoptic‐Scale Precursors of Extreme U.K. Summer 3‐Hourly Rainfall

**DOI:** 10.1029/2018JD029664

**Published:** 2019-04-29

**Authors:** Adrian J. Champion, Stephen Blenkinsop, Xiao‐Feng Li, Hayley J. Fowler

**Affiliations:** ^1^ Department of Meteorology University of Reading Reading UK; ^2^ College of Engineering, Mathematical and Physical Sciences University of Exeter Exeter UK; ^3^ Water Resource Systems Research Laboratory, School of Engineering Newcastle University Newcastle upon Tyne UK

**Keywords:** synoptic meteorology, extreme rainfall, precursors

## Abstract

The synoptic‐scale meteorological conditions leading up to the 30 most extreme subdaily summer rain events for two regions of the United Kingdom (northwest and southeast) were examined for the period 1979–2013. Using a recently available, quality controlled, national hourly rain gauge data set, we were able to identify extreme 3‐hr rainfall accumulations that may be indicative of flash flooding. Composites of the state of the atmosphere leading up to these dates were produced to investigate synoptic‐scale processes, thus potentially allowing for them to be identified in coarse resolution reanalyses and in climate models. The results show that the two regions have different dominant synoptic‐scale conditions leading to extreme 3‐hr rainfall, which is thought to be related to the type of rainfall typically experienced in each region. In particular, positive anomalies in mean sea level pressure and the geopotential height at 200 hPa over the United Kingdom are associated with extreme rainfall in the northwest, where the position of the westerly jet is also important. For the southeast, no clear anomalous synoptic‐scale conditions could be identified; however, localized moisture sources and unstable air masses were observed in association with extremes. These results indicate the importance of better understanding of both synoptic‐scale and thermodynamic drivers of short‐duration extreme rainfall, with potential implications in forecasting and flood warning, as well as for understanding the representation of key processes by regional climate models.

## Key Points

- Different synoptic‐scale meteorological processes dominate on days with JJA subdaily extreme rainfall depending on the region of interest
- A positive anomaly in the geopotential height is associated with extreme rain events in the northwest of the United Kingdom
- Localized moisture sources and unstable air masses observed with extreme rain events in the southeast of the United Kingdom

## 1 Introduction

A warming climate is likely to lead to an intensification of heavy rainfall (Allan & Soden, 2008), which may in turn lead to an increased risk of flash flooding. Such intensification is associated with the water holding capacity of the atmosphere, and extensive research has compared changes in rainfall extremes with the theoretical thermodynamic response of ∼ 6.5%/°C—termed the Clausius‐Clapeyron (CC) rate (Allen & Ingram, 2002; Fischer & Knutti, 2016; Lenderink & van Meijgaard, 2008; Trenberth et al., 2003). However, Lenderink et al. (2017) hypothesized that scaling in excess of CC may be observed but only when the large‐scale atmospheric circulation provides sufficient moisture, noting that intense rainfall events over the Netherlands were typically associated with a convergence of moist air; this is supported by Lochbihler et al. (2017) in their analysis of radar data. A greater effort is therefore needed to understand changes in large‐scale dynamics of the atmosphere and their linkages to atmospheric processes and extreme rainfall (Lenderink & Fowler, 2017; Pfahl et al., 2017).

The costs of extreme weather events, such as storms and floods, are projected to increase with global warming (Stern, 2007), including in the United Kingdom, which experiences a number of summer flash flooding events each year, affecting very localized areas but causing severe disruption and loss (Archer & Fowler, 2015). Further, although it cannot be assumed that extreme subdaily rainfall will always lead to flash flooding, state‐of‐the‐art climate model simulations have projected an increase in the occurrence of intense, short‐duration rainfall for the United Kingdom under a warming climate (Chan et al., 2018; Kendon et al., 2014). Monitoring and recording of these events is however difficult due to their localized nature. Also, the lack of extensive, ground‐based rainfall observations on these time scales is a further constraint on identifying and understanding these events. However, a recent data set of quality controlled, hourly rainfall accumulations for the United Kingdom (Blenkinsop et al., 2017) is enabling improved understanding of subdaily rainfall extremes (Darwish et al., 2018).

The synoptic‐scale atmospheric processes leading up to intense rainfall and flash flooding events have been investigated in a number of countries. Santos et al. (2017) used weather types to investigate flash flooding across three river basins in Portugal, although the study was not limited to the summer, while Barbero et al. (2018) found that disturbances associated with the jet stream and cutoff upper‐level lows were related to the occurrence of 1‐hr annual maximum precipitation across much of the United States. Summer flash flood studies in the United Kingdom have generally focused either on individual case studies or convective‐scale processes (e.g., Flack et al., 2016; Golding et al., 2006; Warren et al., 2013). Although intense U.K. hourly rainfall has been shown to increase with temperature at a rate approximating CC scaling (Blenkinsop et al., 2015; Chan et al., 2016) with some evidence of the sensitivity of this scaling to the prevailing synoptic circulation (Blenkinsop et al., 2015), to date there has been no detailed examination of the synoptic‐scale drivers of intense U.K. subdaily rainfall. Such understanding could contribute to the current broader international research effort to better understand subdaily extreme rainfall and how it might change in a warming climate (Blenkinsop et al., 2018).

It can be expected that locally, intense events are dominated by short‐lived, small‐scale meteorological (e.g., convective) processes but these events are typically difficult to identify in relatively coarse resolution reanalysis data sets. This is also true for their identification in most climate model simulations due to their coarse resolution and the use of parameterized convection schemes, although this is being addressed by the emerging generation of convection permitting regional climate models (Kendon et al., 2016; Prein et al., 2017). This paper therefore uses data from the U.K. rain gauge network to identify extreme 3‐hr rainfall accumulations over different regions of the United Kingdom as these are indicative of potential flash flood events. By using gauge records obtained from the Blenkinsop et al. (2017) observational data set, an analysis based on a larger number of events may now be undertaken. The spatial patterns of synoptic‐scale atmospheric conditions leading up to, and associated with, these events are examined, using a composite approach to identify potential precursor signals in the synoptic conditions, atmospheric stability, and moisture availability fields.

## 2 Data and Method

### 2.1 Atmospheric Data

The daily data from ERA‐Interim (Dee et al., 2011) were used to investigate the synoptic meteorology. ERA‐Interim is a high‐resolution (0.7° latitude by 0.7° longitude) reanalysis product from the European Centre for Medium‐Range Weather Forecasts that spans from 1979 to the present day; the period up until the end of 2013 was used in this study. Daily data were used in preference to the 6‐hourly data as this study focuses on the processes in the days, rather than hours, leading up to extreme rainfall accumulations. The aim is to identify the synoptic‐scale processes that may lead to conditions that could result in more localized mesoscale processes being triggered.

### 2.2 Extreme Rainfall Event Identification

We use a recently available, quality controlled U.K. hourly rainfall data set (Blenkinsop et al., 2017), which is accumulated to 3‐hr totals as this is the operational indicator used to warn of flash flooding in the United Kingdom (Hurford et al., 2012). We then apply a peaks‐over‐threshold n‐largest method to identify individual extremes. This takes the top n events for a given rain gauge (or in this case, region), where here n = 30. In contrast with the annual maxima approach, this potentially allows for multiple events to be identified in a wet year with no events consequently being identified in relatively dry years. The use of this data set allows for the identification of subdaily rainfall extremes at a national scale with a relatively high network density which has previously not been possible. This increases the likelihood of capturing those events that lead to flash flooding whereas daily rainfall extremes, for which data has long existed, would more typically lead to fluvial flooding.

It was expected that the atmospheric conditions associated with extreme rainfall events would be dependent on location (as for U.K. rainfall on daily time scales; e.g., Maraun et al., 2011, 2012) and so rain gauges/extreme accumulations were categorized using the regionalization of Jones et al. (2013). Northwest England (NW) and southern England (SE) were selected for comparison as these regions are characterized by differences in topography and different positions in relation to moisture sources: The NW region is dominated by high (in U.K. terms) orography, lies on the Irish Sea coast, and is more exposed to storms traveling across the Atlantic Ocean, whereas the SE region is predominantly flat and less directly influenced by the dominant westerlies compared to the NW.

**Figure 1:**
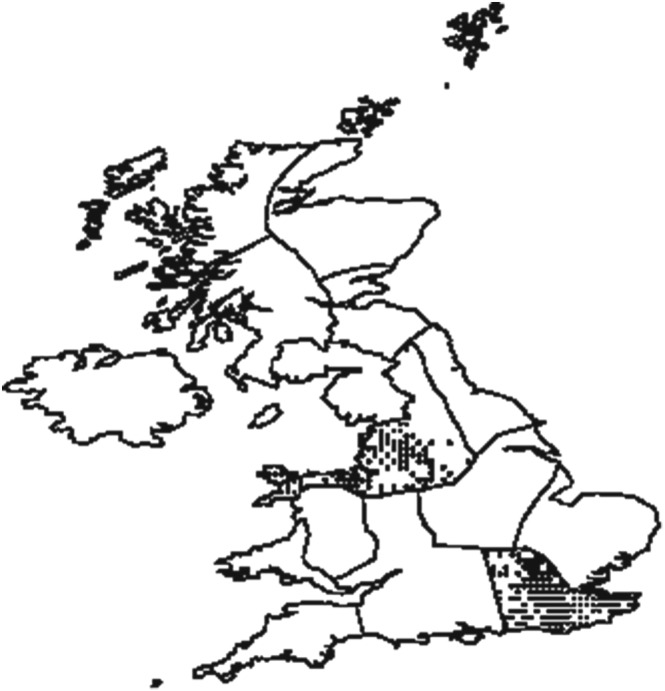
Location of the rain gauges in the two extreme rainfall regions used in the case study: the top left locations, 153 rain gauges in northwest England (NW), which is dominated by areas of relatively high orography; the bottom right locations, 217 rain gauges in southern England (SE), which is relatively flat.

Extreme rain events in NW may therefore be dominated by orographically enhanced processes compared to events in SE, which has less orography and may therefore be influenced more by summer convective processes (Blenkinsop et al., 2017). Thus, the synoptic‐scale conditions leading up to extreme rain events were compared for these two contrasting regions to identify any differences or alternatively determine whether only differences in mescoscale processes (processes of the order of hundreds of kilometers in size) could be identified. For the NW region 153 gauges were used to identify the extreme rainfall accumulations, and for the SE region 217 gauges were used; these are shown in Figure 1. It should be noted that there are few rain gauges from the upland Pennines used in this study due to the relative scarcity of gauges at higher elevations and as a consequence of the exclusion of those that are of relatively short duration or included a high proportion of data identified as erroneous by the quality control process (Blenkinsop et al., 2017). Thus, the topographic effect of the NW may not be fully sampled. The southwest region of Scotland was not used in this study for similar reasons. For each region the 30 largest summer (June–August) 3‐hr rainfall accumulations were identified (Table 1); note that to ensure independence, events were defined as occurring on separate calendar days.

**Table 1:** Dates, Intensities, and Location of the Rain Gauges of the Case Studies Investigated for Each Extreme Rainfall Region, Ranked by Intensity for Each Region

Southern England				Northwest England			
Date	Intensity (mm/hr)	Latitude	Longitude	Date	Intensity (mm/hr)	Latitude	Longitude
15‐08‐2010	104.8	51.7	359.7	28‐08‐1990	140.0	53.7	357.8
23‐07‐2012	100.0	51.5	359.7	06‐08‐1981	73.0	53.4	357.7
10‐06‐1993	79.2	51.7	0.1	03‐08‐2004	64.8	53.7	357.8
27‐06‐1994	76.4	51.1	0.7	22‐07‐2010	62.8	53.2	356.0
15‐07‐2008	74.2	51.2	359.6	02‐07‐2006	62.0	53.7	357.8
06‐07‐1983	73.7	51.5	359.7	30‐08‐2009	59.4	53.0	355.9
07‐07‐2009	70.2	50.9	0.4	02‐07‐2006	57.2	53.7	357.8
04‐06‐2005	69.6	51.3	0.0	20‐08‐2010	51.6	53.0	355.9
05‐08‐1994	67.8	51.2	0.4	31‐08‐1997	51.5	53.3	358.4
07‐07‐2001	66.6	51.0	359.3	17‐07‐2003	49.6	53.9	357.8
07‐08‐2002	66.5	51.6	359.8	28‐07‐2013	48.6	53.9	357.7
20‐08‐2002	64.4	51.4	0.0	28‐06‐1995	48.4	53.2	356.4
04‐07‐2000	59.6	51.6	359.8	06‐08‐2007	48.0	53.8	358.1
15‐06‐1998	58.8	51.4	0.0	03‐07‐2001	46.6	53.1	356.0
13‐08‐2006	58.6	50.9	359.5	30‐07‐2002	44.2	53.8	357.7
20‐07‐2007	58.4	51.3	0.7	27‐06‐2009	43.8	53.3	356.2
20‐08‐2005	58.3	51.1	359.5	03‐08‐2005	43.6	53.6	357.9
19‐06‐2007	57.2	51.3	359.4	19‐06‐2005	43.6	53.8	357.6
05‐07‐1999	56.2	51.0	359.3	24‐06‐1994	43.3	53.6	357.8
06‐08‐1997	55.8	51.3	359.9	22‐07‐2007	43.0	52.9	355.6
04‐08‐1999	55.4	51.3	359.4	14‐06‐2002	42.7	53.2	357.0
23‐07‐1996	55.2	51.0	359.9	31‐07‐1998	42.6	53.2	356.9
08‐07‐1997	55.2	51.4	359.8	13‐06‐2007	42.5	53.2	358.1
10‐08‐1994	54.8	51.1	1.2	07‐07‐2008	42.2	53.0	355.8
11‐06‐2012	54.6	51.2	0.8	01‐08‐1995	42.2	53.6	357.9
03‐06‐2012	53.4	51.3	359.8	20‐08‐2004	42.0	53.6	357.6
13‐08‐2010	53.4	50.9	0.1	03‐07‐2000	41.8	54.5	357.8
21‐08‐2007	53.0	51.1	1.2	24‐08‐2005	41.4	53.0	356.9
08‐08‐1999	52.8	50.9	359.5	17‐08‐2008	41.0	53.0	355.9
27‐07‐2006	52.4	51.9	359.7	12‐08‐2004	41.0	53.0	357.0

## 3 Results

The focus of this study is on the larger‐scale atmospheric conditions and processes that can be identified in reanalysis data and potentially in coarse‐resolution global climate models rather than investigating the convective‐scale processes that are known to trigger summer extreme rainfall events. However, the identification of atmospheric conditions that result in a moist, unstable atmospheric state, which, when triggered by mesoscale processes, are likely to result in extreme rain, would provide a significant improvement in understanding and potentially in forecasting such events (and thus flood warning).

The investigation looks at composites of the 30 highest 3‐hr accumulations for each region. The composites shown in this section are the anomalies of the daily means of each field to the monthly mean, with the composites of the absolute daily means shown in the supporting information (Figures S1 and S2). Anomalies were calculated based on monthly, seasonal, and climatological means; however, only those relative to the monthly mean are shown here as they show the largest correlation to the extreme rainfall accumulations examined. It was not possible to calculate a significance value for each anomaly; however, we have only discussed features where the absolute magnitude of the anomalies is greater than the range seen in the spread of the 30 events that make up the composites.

The results from the investigation into the synoptic situation, the mean sea level pressure (MSLP), and relative humidity at 850hPa are looked at first (section 3.1). The stability criteria are then examined in section 3.2 followed by the geopotential height in section 3.3. We then move on to the moisture availability and transport variables (vertically integrated horizontal water vapor transport [IVT] and total column water vapor [TCWV]) in section 3.4, before finally looking the relationship of the extreme events with the westerly jet in section 3.5.

### 3.1 Synoptic Conditions

**Figure 2:**
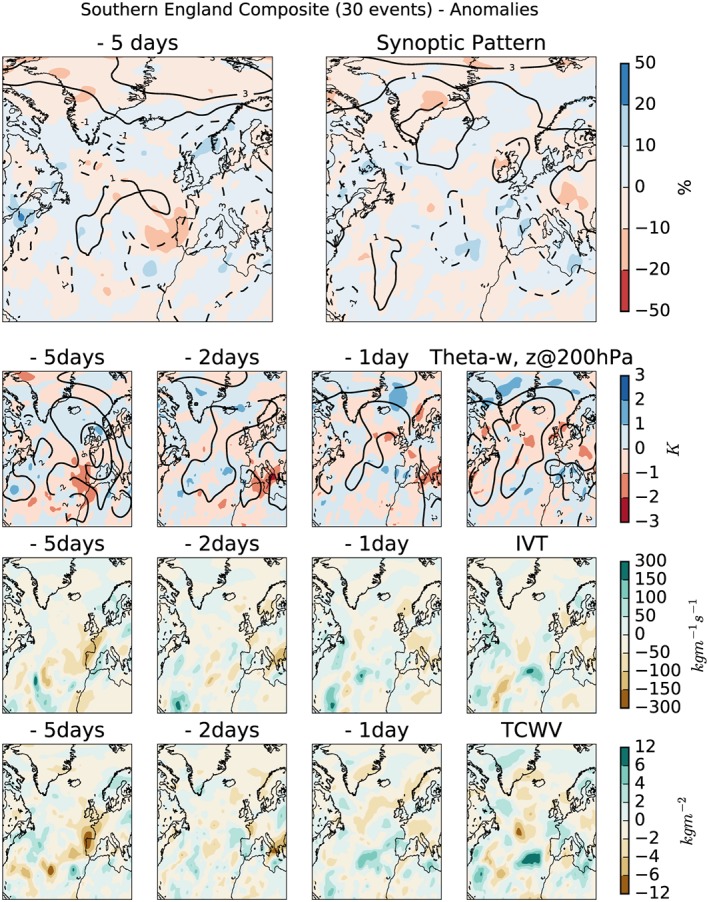
Anomalies with respect to the monthly average of the selected metrics discussed in section 3 for the 30 most extreme 3‐hr rain events identified in the southern region. (top row) The “synoptic pattern,” mean sea level pressure (isobars, negative anomalies shown as dashed lines), relative humidity at 850 hPa (shading); (second row) geopoential height (z) at 200 hPa (isolines), difference in the θ_w_ between 850 and 500 hPa (shading); (third row) vertically integrated horizontal water vapor transport; (bottom row) total column water vapor. IVT = integrated horizontal water vapor transport; TCWV = total column water vapor.

**Figure 3:**
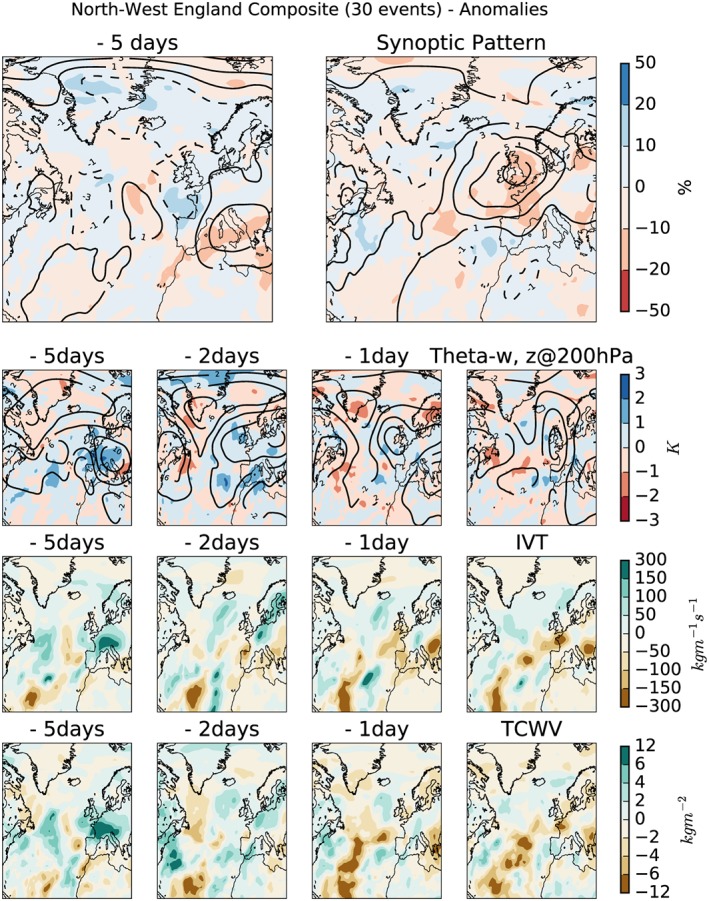
Anomalies with respect to the monthly average of the selected metrics discussed in section 3 for the 30 most extreme 3‐hr rain events identified in the northwest region. (top row) The “synoptic pattern,” mean sea level pressure (isobars, negative anomalies shown as dashed lines), relative humidity at 850 hPa (shading); (second row) geopoential height (z) at 200 hPa (isolines), difference in the θ_w_ between 850 and 500 hPa (shading); (third row) vertically integrated horizontal water vapor transport; (bottom row) total column water vapor. IVT = integrated horizontal water vapor transport; TCWV = total column water vapor.

Composites of the daily anomalies (MSLP, isobars, negative anomalies shown as dashed lines), with respect to the monthly mean, are shown in the top rows of Figures 2 and 3 for SE and NW respectively; the absolute daily means are shown in Figures S1 and S2. Composites are shown for 2 days: the day the extreme rainfall occurred (right) and 5 days prior to its occurrence (left). While low‐pressure systems are common in winter and cause 60% of the winter rainfall over the United Kingdom (Hawcroft et al., 2012), they are not typically associated with extreme summer rain on subdaily time scales.

As would be expected, the patterns in the anomaly plots are relatively noisy (compared to the daily means Figures S1 and S2). The composite for SE (Figure 2) 5 days prior to the extreme rainfall accumulation shows lower pressure, compared to the monthly average, over western Europe. There is also an area of increased pressure over the pole, which persists through to the day of the rainfall. In contrast, the relatively low pressure over western Europe moves south and an area of high pressure develops over the United Kingdom. The relative humidity field has a positive anomaly 5 days prior to the rainfall over the north of the North Sea. This has disappeared by the day of the rain event, although another positive anomaly can be seen to have developed to the southwest of the English Channel.

A very clear positive anomaly is seen in the MSLP for NW (Figure 3) on the day of the extreme rain accumulation, a much clearer signal than can be seen for SE. The signal is very noisy 5 days prior to the rainfall with no clear anomalies, as was also the case for SE. The large negative anomaly in the relative humidity on the day of the rainfall for NW is suggestive that the moisture supply has been used by the rain event. Combined with the large positive anomaly in MSLP, this could suggest the relative dominance of orographically enhanced rain for the northwest of England compared to the SE region.

### 3.2 Stability Criteria

Next we examined the stability of the atmosphere. Typically, these are small‐scale fields or are not widely available in reanalyses therefore surrogates for atmospheric stability/CAPE (Convective Available Potential Energy) were used. Although CAPE is available in the reanalysis used in this study, a surrogate was used so that this work could be applied to other data sets. The surrogate chosen in this study was the wet‐bulb potential temperature, based on Davies‐Jones (2008) and N. Roberts (personal communication, December 18, 2014). To calculate the wet‐bulb temperature surrogate, a dry‐bulb temperature surrogate had to be calculated first:

(1) $$\theta_{e}=(T+2.46\times 10^{3}q){\left(1,000/p\right)}^{0.285},$$

where *T* is the temperature, *q* is the specific humidity, and *p* is the pressure. The wet‐bulb potential temperature (*θ*_*w*_) surrogate was then calculated using the equations from Davies‐Jones (2008):

(2) $$\theta_{w}=45.114-51.489{\left(\theta_{e}/C\right)}^{-\lambda },$$

where *λ*≡1/*κ*_*d*_ ( = 3.504) as *κ*_*d*_ = *R*_*d*_/*c*_*pd*_ ( = 2.2854) is the Poisson constant for dry air, and *C* is a scaling constant =273.15 K.

The stability of the atmosphere can be inferred by taking the difference in the *θ*_*w*_ surrogate values between two levels in the atmosphere. If the stability surrogates are less negative at higher levels (i.e., the difference is negative) that is suggestive that the atmosphere will be unstable—it is representative of an air parcel rising from a warm and moist lower atmosphere to a colder and drier upper atmosphere.

Whilst the moist surrogate, *θ*_*w*_, is used in this paper, results (not shown) did not show much difference between the *θ*_*w*_ and *θ*_*e*_ surrogates. The choice of levels used to calculate the differences in *θ*_*w*_ was also investigated but again found similar values for each pair of levels (850, 700, and 500 hPa); the 850‐ to 500‐hPa difference was chosen as it showed the largest differences. A comparison between the *θ*_*w*_ (850–500hPa) field and CAPE (not presented) showed that a similar pattern, both spatially and the evolution of that pattern over time, is seen in the two fields for both regions. The same conclusions were drawn in regard to the stability/instability of the atmosphere for both fields and whether they were a precursor for the extreme rain accumulations.

The composites of *θ*_*w*_ (850–500 hPa) can be seen in the colored contours on the second row of Figures 2 and 3; areas of instability are shown as areas with negative differences (red) suggesting an unstable atmosphere to moist parcels of air lifted from the lower levels. The results for SE (Figure 2) show that the instabilities seen over the United Kingdom and continental Europe are not unusual (within ±1 K). Thus, it is clear that the stability criterion alone is not indicative of extreme 3‐hr rainfall but needs to be combined with the presence of a moist atmosphere, as we might expect.

A different pattern (and evolution of that pattern) in the anomalies for NW events (Figure 3) is seen over Europe. A positive anomaly is seen over the United Kingdom and western Europe 5 days prior to the extreme rainfall, suggesting the atmosphere is more stable than the monthly average. However, this becomes more similar to the monthly average by the day of the rain event. The evolution of the pattern seems to very closely match the evolution of the geopotential height anomaly, discussed in the next section. As with the synoptic pattern, we conclude here that the processes leading to extreme 3‐hr summer rain over NW are different to those for SE.

### 3.3 Geopotential Height

An issue with the stability criteria is that they tend to be relatively localized, resulting in noisy spatial patterns, as observed in our results. Another variable that is often used to investigate atmospheric instability is the geopotential height. If a low in the geopotential height at a given pressure level is observed, it suggests a height cap, limiting the vertical movement of an air parcel. If this air parcel is moist, this height cap increases the chances of convection being triggered. This is a simplified approach of looking for cutoff lows, which were determined to be present during the U.K. summer floods of 2007 by Blackburn et al. (2008) and are a driver of a large proportion of hourly precipitation extremes in the United States (Barbero et al., 2018). Cutoff lows are identified here using four levels of geopotential height: 200, 300, 400, and 500hPa.

Results (not shown) from an investigation into the sensitivity to the choice of level showed that all four levels identified lows in the same location and at the same time. The 200‐hPa level was therefore chosen and is presented in Figures 2 and 3 as this had the deepest lows and follows the choice used by Nieto et al. (2005) in the identification of cutoff lows. As discussed in the previous section, it is likely that when combined with a measure of atmospheric moisture and/or transport there is a greater correlation to when extreme events occur compared to using the geopotential height in isolation.

The geopotential height at 200‐hPa (z@200hPa) anomaly to the monthly mean is shown as contours on the second row of Figures 2 and 3. For SE (Figure 2) a trough seen 5 days prior to the extreme rainfall is shown to be anomalously low compared to the monthly average; however, it is a very noisy picture over the North Atlantic and western Europe. This anomalous low reduces in magnitude closer to the day of the rain event and moves west, toward North America, as also seen in the composite for absolute geopotential height (Figure S1). As for *θ*_*w*_, both fields become less coherent and smaller in magnitude as the day of the rain event approaches.

A much clearer pattern is seen for NW (Figure 3), which also matches the pattern seen in the *θ*_*w*_ field. The geopotential height is shown to be a large positive anomaly, compared to the monthly mean, over western Europe. While the magnitude of this anomaly decreases toward the day of the extreme rainfall, the extent of the anomaly also decreases but moves to and remains over the United Kingdom, with a positive anomaly being seen over the United Kingdom on the day of the rainfall. This corresponds to the ridge in absolute geopotential height seen in Figure S2.

These anomalies in the geopotential height are likely related to the location of the storm track; a positive anomaly in the geopotential height field suggests strong storm track activity. However, while the same general conclusion may be inferred as before, that the type of processes dominating the extreme rain event in the two regions differs, the reason for the opposing evolution in both geopotential height anomaly and the *θ*_*w*_ anomaly for the two regions is not clear.

### 3.4 Moisture Availability and Transport

The previous sections have highlighted the importance of looking at moisture availability and moisture transport. In this study two variables were investigated—IVT and TCWV. Potentially, by looking at these variables in combination with one of the stability variables, this will provide information as to when the atmosphere is both moist and unstable thus increasing the chances of convection being triggered. IVT is used in the identification of atmospheric rivers (ARs), which have also been shown to be associated with winter flooding (Lavers et al., 2011), although previous work has also indicated that they are not associated with daily summer extreme rain (Champion et al., 2015). Here, IVT is not used to identify ARs but used as a stand alone field (third row of Figures 2 and 3) along with the TCWV field (bottom row).

#### 3.4.1 IVT

Figure 2 shows that for SE the IVT field is not anomalous, compared to the monthly mean, with very small magnitudes seen, which matches the pattern observed for the absolute values (Figure S1). This is not very surprising given the results from the summer AR study by Champion et al. (2015). However, for NW (Figure 3), the area of high IVT seen over France is shown to be anomalous; the IVT is higher compared to the monthly mean. This becomes a large negative anomaly (showing low IVT) on the day of the rain event, suggesting that it provided the moisture supply for the extreme rainfall. While this seemingly contradicts the previous statement about the Champion et al. (2015) study, it again highlights the different processes dominant in the two regions. This again suggests the importance of orographic enhanced rain in NW compared to SE.

#### 3.4.2 TCWV

While the absolute values of TCWV are low (Figures S1 and S2), there are areas that can be observed to be anomalous compared to the monthly mean. For SE (Figure 2) the TCWV is shown to be relatively low over the Bay of Biscay 5 days prior to the extreme rainfall. This region becomes more similar to the monthly mean by the day of the rainfall. However, as for IVT, NW (Figure 3) shows a different pattern. There is a large positive anomaly (high TCWV) over France 5 days prior to extreme rainfall which becomes a negative anomaly on the day of the rainfall. It is perhaps not surprising that this pattern and trend (for both the NW and SE) is the same as observed for IVT.

These contrasting behaviors are similar to those seen for θ_w_ and geopotential height. The results presented in this section suggest that there is greater moisture available in the immediate vicinity of the United Kingdom and leading up to NW region extreme rainfall, compared to both the monthly average and to SE region extremes. It is possible, using the original hypothesis of the type of rainfall experienced, that NW events are dominated by synoptic‐scale orographic forcing and reliant on large sources of moisture. For SE, in comparison, extreme 3‐hr rainfall may be more dependent on organized cells of convection, although this has not been shown explicitly here. However, to not see any local sources of moisture for the suggested convective cells is puzzling.

### 3.5 Relationship With The Westerly Jet

**Figure 4:**
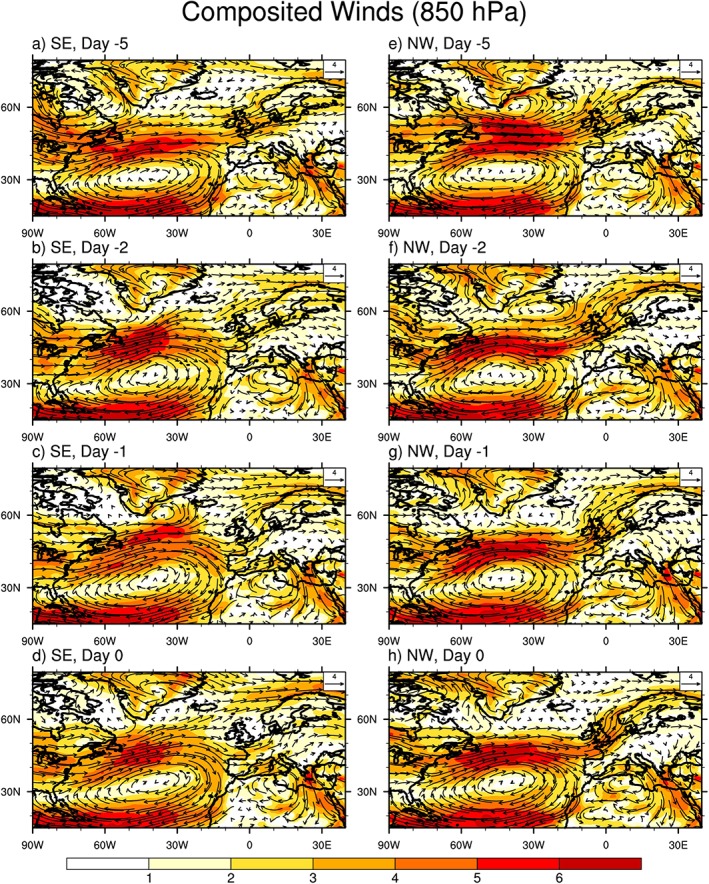
Composited horizontal wind vectors (m/s) and absolute wind speed (color shaded, m/s) at 850 hPa for the 30 most extreme rainfall accumulations over each region. From 5 days before the extreme events rainfall (marked as −5) to the day of extreme events rainfall (marked as 0) over (a–d) SE and over (e–h) NW. Wind vector length scale is in the upper right corner of panels. NW = northwest England; SE = southern England.

During the summer the Azores high dominates the southern part of the Northern Atlantic, centered at 40°W, 35°N, which has a strong impact on the surrounding inland rainfall (Davis et al., 1997; Li, Li, Kushnir, et al., 2012; Li, Li, Ting, et al., 2012). The United Kingdom is located at the downstream end of the westerly jet, which is associated with the northern branch of the Azores high in the lower troposphere. Hence, the Azores high has a strong influence on the circulation pattern over the United Kingdom. Analysis of the ERA‐Interim horizontal wind vector and wind speed fields indicated that the position of the westerly jet associated with the Azores high in the midlatitudes over the United Kingdom is very different during the extreme 3‐hr rainfall for the NW and SE (Figure 4).

**Figure 5:**
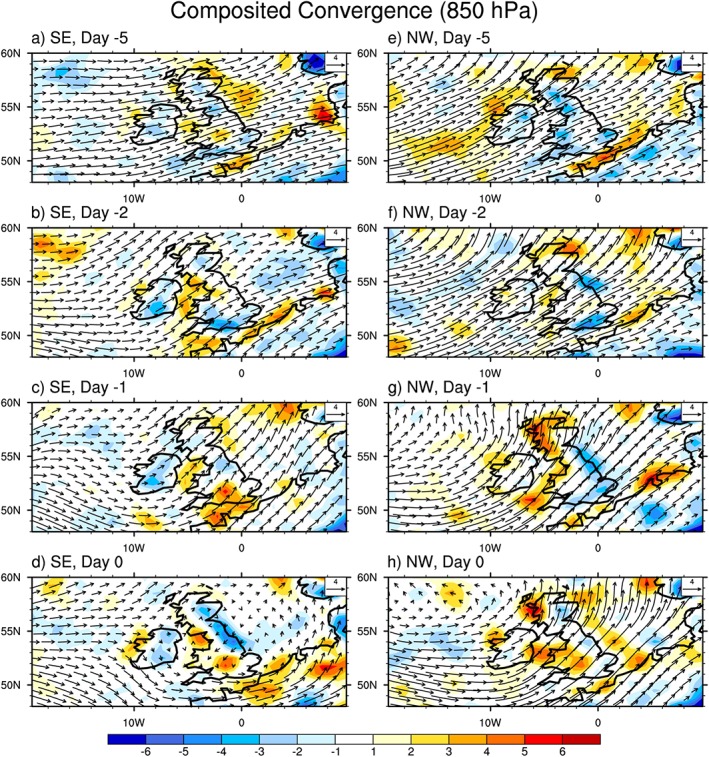
Composited horizontal wind vectors (m/s) and absolute horizontal convergence (color shaded,10^−5^ g·kg^−1^·s^−1^) at 850 hPa for the 30 most extreme rainfall accumulations over each region. From 5 days before the extreme rainfall (marked as −5) to the day of extreme rainfall (marked as 0) over (a–d) SE and over (e–h) NW. Wind vector length scale is in the upper right corner of panels. NW = northwest England; SE = southern England.

For SE extreme rainfall (Figure 4d), the westerly jet is located to the south of the United Kingdom resulting in a cyclonic circulation over the SE region on the day of extreme rainfall. This is consistent with the weak horizontal wind over the region, as verified by the daily averaged absolute wind being less than 2 m/s. However, atmospheric convergence (Figures 4d and 5d) in the lower troposphere is observed at the same time, which is greater than for surrounding areas, as evidenced by the convergence being more than 5 × 10^−5^ g·kg^−1^·s^−1^ over the region on the day of extreme rainfall. It is known that horizontal convergence in the lower troposphere and upward motions in the midtroposphere usually occur in the central location of a cyclonic circulation, which is located over the SE region in this case. This suggests that the 3‐hr extreme rainfall accumulations over SE are dominated by convective processes.

In contrast, for NW (Figures 4e–4h and 5e–5h), the westerly jet is located over the United Kingdom, resulting in strong westerlies across the region, with daily averaged absolute wind speed over 4 m/s, double that observed for SE extreme rainfall. As the NW region faces the Atlantic westerlies and has higher elevation relative to SE (not shown), the lifting effect of the topography on moist westerlies is an important causal mechanism for extreme rainfall over this region. Coincident with the lifting effects, convergence is also observed in the lower troposphere over NW (Figure 5h) that is usually associated with upward motions according to the continuity of mass law. Given that the Atlantic westerlies are highly related to the Azores high in summer, we believe NW extreme rainfall events are dominated by large‐scale processes, consistent with the discussion in section 3.4. We also note that a low‐level trough emerges over the United Kingdom from 2 days before the event onward (Figures 4f–4h), which could also contribute to the triggering of extreme rainfall events over this region but this requires further investigation. Further, the question of whether the formation of the low‐level cyclonic circulation is the critical component to trigger extreme rainfall over northwest England also needs further investigation but these are out of the scope of the current study.

## 4 Conclusions

This study used a recently available, quality controlled hourly rain gauge data set (Blenkinsop et al., 2017) to select extreme 3‐hr rainfall accumulations for northwest England (NW) and southern England (SE) in the United Kingdom. These two regions were chosen due to their contrasting topography and exposure to westerlies from the Atlantic Ocean. The top 30 events for each region were identified and composites of the synoptic‐scale atmospheric conditions were created from ERA‐Interim reanalysis data.

We demonstrate that the “synoptic pattern” is not unusual in the days leading up to the extreme rainfall. However, for NW, on the day of the rain event a positive anomaly (high pressure), relative to the monthly mean, in the MSLP is seen centered over the United Kingdom; no clear anomaly is seen for SE. The relative humidity field shows a small region of high relative humidity in the days preceding the rain event, for both SE and NW, but only NW sees a negative anomaly on the day of the extreme rainfall, suggesting that the local moisture supply has been used by the process that led to the rain event for the NW.

The *θ*_*w*_ field indicates an unstable atmosphere over the United Kingdom and its surrounding region on the day of the extreme rainfall and for the preceding 5 days. However, compared to the monthly mean, this instability was not anomalous for SE. A positive anomaly was observed for NW 5 days prior to the extreme rainfall, but this was not anomalous by the day of the rainfall. The pattern and evolution of the geopotential height at 200 hPa was similar to those for *θ*_*w*_ for both regions. No clear signal in the geopotential height could be seen for SE. However, the geopotential height had a positive anomaly over western Europe 5 days prior to extreme rainfall for NW, which decreased in magnitude and centered over the United Kingdom by the day of the rainfall. The signal in the geopotential height was also thought to be related to storm track activity, with the positive anomaly suggesting high storm track activity.

The *θ*_*w*_, geopotential height, moisture availability, and convergence variables (IVT and TCWV) are all dynamically linked, and thus, it is not surprising that they show similar trends and patterns. A positive anomaly in the geopotential height indicates a ridge; a high TCWV indicates a moist atmosphere. This suggests there is upward motion of moist air within the atmosphere, which will result in high *θ*_*w*_. Further, the IVT field indicates areas of moisture convergence, which explains the colocation with areas of high *θ*_*w*_. The benefit of using geopoential height and TCWV is that they are larger‐scale fields, compared to *θ*_*w*_, which makes them easier to identify in coarse resolution models.

The strong, moist westerly jet associated with the Azores high, combined with the high topography in the NW region has also been shown to be associated with the occurrence of extreme rainfall. While the SE region has less topography, the effect of the westerly jet when over the southern United Kingdom is to create a cyclonic circulation resulting in strong upward motions over the region, a key condition for the development of convective events.

A number of conclusions can be drawn from these results. We show that the *θ*_*w*_ field, a localized field, which is an indicator of convection, can be inferred from larger‐scale fields—positive anomalies in both the geopotential height and TCWV. We also show that the processes leading to extreme subdaily rainfall are dependent on the region, using two contrasting regions for the United Kingdom. The NW region, due to the presence of orography, is strongly affected by larger‐scale orographically enhanced processes that are less reliant on localized convection. The SE region has more localized sources of moisture and has relatively unstable air masses leading up to extreme rainfall. While we believe that SE extremes are driven more by convective processes than large‐scale processes (as shown for NW), this cannot be proven here and requires further investigation, outside of the scope of this paper.

These results are consistent with those derived from simulations of hourly rainfall from models, including those at a 1.5‐km convection permitting scale, indicating that larger‐scale conditions are important for the generation of subdaily extreme rainfall. Chan et al. (2018) found that stability and to a lesser degree relative vorticity and MSLP displayed some skill as predictors of hourly extremes in simulations over the southern half of the United Kingdom. These results, combined with our observational analysis, indicate that the role of large‐scale conditions must be considered alongside potential thermodynamic effects when providing projections of changes in extreme short‐duration rainfall. Additional benefits for the climate modeling community include better understanding of model process deficiencies, which could lead to improved skill in the simulation of extreme rainfall by climate models. Further, the skill of large‐scale variables as predictors of the potential occurrence of intense rainfall could facilitate a more intelligent use of high‐resolution convection permitting models to simulate only periods with a high likelihood of extremes, thus making larger ensembles more feasible.

This study confirms that the availability of quality controlled rainfall observations is essential to improve our understanding of subdaily extremes. Further, the influence of the large‐scale atmospheric circulation needs to be examined in conjunction with local thermodynamics to more fully understand the drivers of these extremes (Lenderink & Fowler, 2017; Pfahl et al., 2017). These issues are being addressed at the global scale by the INTENSE (INTElligent use of climate models for adaptatioN to non‐Stationary hydrological Extremes) project (Blenkinsop et al., 2018), which leads the global research effort within the Global Energy and Water Exchanges (GEWEX) Hydroclimatology Panel Cross‐Cutting project on subdaily precipitation extremes, addressing the World Climate Research Programme “Grand Challenge” on extremes. In particular, a better understanding of the large‐scale precursors to extreme short‐duration rainfall could then lead to improved projections of climate change from climate models, which could in turn lead to long‐term improvements in the preparedness for the impacts of intense rainfall and flash flooding. This is especially important for the provision of guidance on estimates of change in rainfall intensity for urban water drainage (Dale et al., 2017). On shorter time scales, such knowledge could also contribute to better short‐term forecasting of flash flooding from intense rainfall, potentially reducing loss of life and economic costs.

## Supporting information



Supporting Information S1

Figure S1

Figure S2
