# Supplementary material for: Synoptic‐Scale Precursors of Extreme U.K. Summer 3‐Hourly Rainfall
Source: J Geophys Res Atmos. 2019 Apr 29;124(8):4477–89. doi: 10.1029/2018JD029664 (PMC6582617; doi:10.1029/2018JD029664)
Supplement: Supplementary file 1 — Supporting Information S1 [file JGRD-124-4477-s001.pdf]

Adrian J. Champion[1,2], Stephen Blenkinsop[3], Xiao-Feng Li[3], Hayley J. Fowler[3]

[1]Department of Meteorology, University of Reading, Reading, UK.

[2]College of Engineering, Mathematical and Physical Sciences, University of Exeter, Exeter, UK.

[3]Water Resource Systems Research Laboratory, School of Engineering, Newcastle University, UK.

Contents

1. Figures S1 to S2

Additional Supporting Information (Files uploaded separately)

1. Captions for Figures S1 to S2

Introduction

Figure S1.

The absolute values of the selected metrics discussed in Section **??** for the 30 most extreme 3h rain events identified in the southern region. Top row: the 'synoptic pattern', MSLP (isobars), relative humidity at 850hPa (shading); 2nd row: geopoential height (z) at 200hPa (isolines), difference in the θ_w between 850hPa and 500hPa (shading); 3rd row: vertically integrated horizontal water vapour transport; bottom row: total column water vapour.

Figure S2.

The absolute values of the selected metrics discussed in Section **??** for the 30 most extreme 3h rain events identified in the north-west region. Top row: the 'synoptic pattern', MSLP (isobars), relative humidity at 850hPa (shading); 2nd row: geopoential height (z) at 200hPa (isolines), difference in the θ_w between 850hPa and 500hPa (shading); 3rd row: vertically integrated horizontal water vapour transport; bottom row: total column water vapour.

Corresponding author: Adrian J. Champion, `a.champion@exeter.ac.uk`
