# Supplementary material for: Synoptic‐Scale Precursors of Extreme U.K. Summer 3‐Hourly Rainfall
Source: J Geophys Res Atmos. 2019 Apr 29;124(8):4477–89. doi: 10.1029/2018JD029664 (PMC6582617; doi:10.1029/2018JD029664)
Supplement: Supplementary file 2 — Figure S1 [file JGRD-124-4477-s002.pdf]

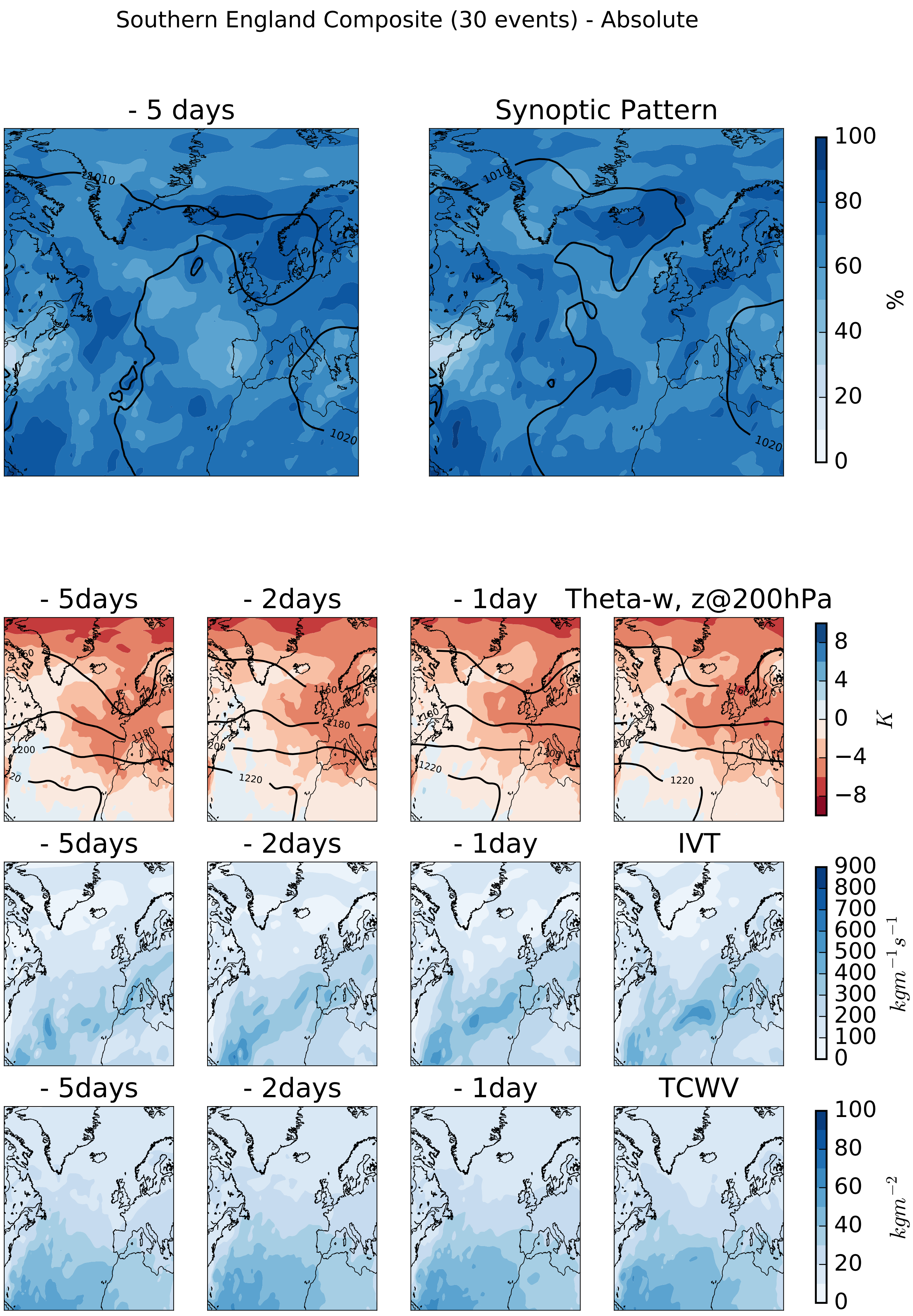
Southern England Composite (30 events) - Absolute
- 5 days
Synoptic Pattern
1010
1020
100
80
60
40
20
0
%
- 5days
- 2days
- 1day
Theta-w, z@200hPa
1160
1180
1200
1220
8
4
0
−4
−8
K
- 5days
- 2days
- 1day
IVT
900
800
700
600
500
400
300
200
100
0
$kgm^{-1}s^{-1}$
- 5days
- 2days
- 1day
TCWV
100
80
60
40
20
0
kgm^{-2}
