# Supplementary material for: Synoptic‐Scale Precursors of Extreme U.K. Summer 3‐Hourly Rainfall
Source: J Geophys Res Atmos. 2019 Apr 29;124(8):4477–89. doi: 10.1029/2018JD029664 (PMC6582617; doi:10.1029/2018JD029664)

North-West England Composite (30 events) - Absolute

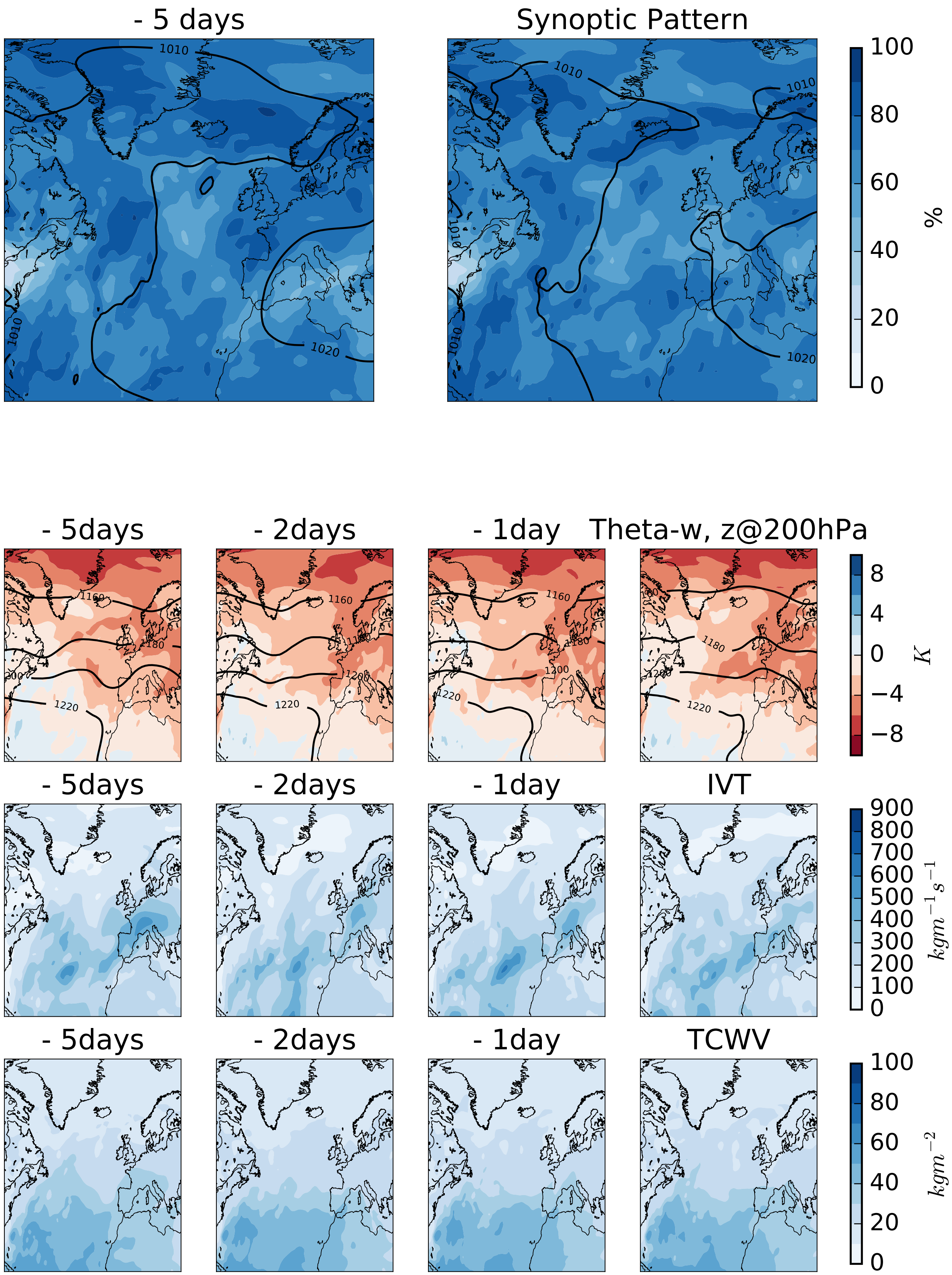

Supplement: Supplementary file 3 — Figure S2 [file JGRD-124-4477-s003.pdf]
